# A novel monoclonal antibody with improved FcγR blocking ability demonstrated non-inferior efficacy compared to IVIG in cynomolgus monkey ITP model at considerably lower dose

**DOI:** 10.1093/cei/uxac112

**Published:** 2022-12-08

**Authors:** Yuko Nakajima-Kato, Masato Komai, Tadashi Yoshida, Akiko Kanai

**Affiliations:** Biomedical Science Research Laboratories 2, Research Unit, R&D Division, Kyowa Kirin Co., Ltd., Japan; Graduate School of Bio-Applications and Systems Engineering, Tokyo University of Agriculture and Technology, Tokyo, Japan; Biomedical Science Research Laboratories 2, Research Unit, R&D Division, Kyowa Kirin Co., Ltd., Japan; Graduate School of Bio-Applications and Systems Engineering, Tokyo University of Agriculture and Technology, Tokyo, Japan; Department of Applied Biological Science, Tokyo University of Agriculture and Technology, Tokyo, Japan; Biomedical Science Research Laboratories 1, Research Unit, R&D Division, Kyowa Kirin Co., Ltd., Japan

**Keywords:** FcγRs, ITP, IVIG, platelets, cynomolgus monkey model

## Abstract

Intravenous immunoglobulin (IVIG) is a well-established treatment for various autoimmune and inflammatory diseases. However, the standard dose prescribed for autoimmune diseases, including immune thrombocytopenic purpura (ITP), is 2 g/kg, which is markedly high and leads to a high treatment burden. In this study, we generated fragment crystallizable (Fc)-modified anti-haptoglobin (Hp) monoclonal antibodies with non-inferior efficacy compared to IVIG at considerably lower doses than IVIG, as shown by *in vitro* experiments. We evaluated binding activity of anti-Hp antibodies to Fc gamma receptors (FcγRs) with ELISA and inhibitory activity against the ADCC reaction. Furthermore, we successfully established a novel cynomolgus monkey ITP model and demonstrated that the anti-Hp antibody exerted its effect in this model with only a single dose. This Fc-modified anti-Hp monoclonal antibody could be a valuable therapeutic replacement for IVIG for the treatment of ITP.

## Teaser

We generated fragment crystallizable (Fc)-modified anti-haptoglobin (Hp) monoclonal antibodies with non-inferior efficacy compared to IVIG at considerably lower doses than IVIG and evaluated binding activity of anti-Hp antibodies to Fc gamma receptors with ELISA and inhibitory activity against the ADCC reaction. Furthermore, we successfully established a novel cynomolgus monkey ITP model and demonstrated that the anti-Hp antibody exerted its effect in this model with only a single dose. This Fc-modified anti-Hp monoclonal antibody could be a valuable therapeutic replacement for IVIG for the treatment of ITP.

## Graphical Abstract

**Graphical Abstract:**
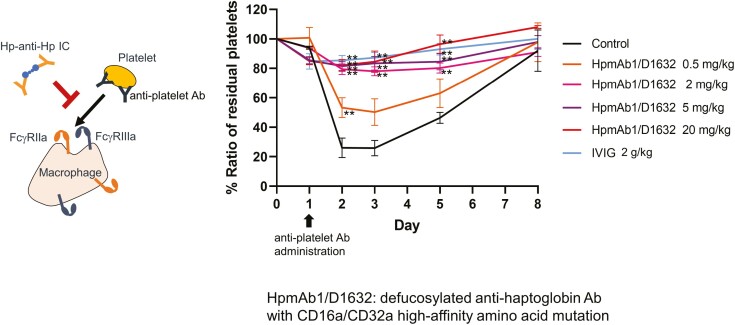

## Introduction

Immune thrombocytopenic purpura (ITP) is an autoimmune disease that is primarily caused by macrophage-mediated phagocytosis of autoantibody-opsonized platelets in the reticuloendothelial system. Intravenous immunoglobulin (IVIG) exerts its immunomodulatory effects by blocking fragment crystallizable (Fc) gamma receptors (FcγRs), including FcγRIIa (CD32a) and FcγRIIIa (CD16a), in ITP [1–4]. Although the mechanisms of action of IVIG have not been completely elucidated, the results from the clinical trial of GMA161, an anti-CD16 antibody that has shown efficacy on thrombocytopenia improvement in ITP patients [5], suggest that blockade of FcγRs is a possible mechanism of IVIG action.

Low-affinity FcγRs, including CD16a and CD32a, bind immune complexes (ICs) with remarkably higher affinity than immunoglobulin G (IgG) monomers [1]. In this context, inhibition of FcγRs by IVIG has been proposed to be further enhanced by the formation of ICs between the antibodies contained in IVIG and some components in the serum and by IgG dimers in the formulation [1, 6]. Indeed, Lemieux and others studies have shown that autoreactive IgGs isolated from IVIG form soluble ICs when mixed with serum [7, 8]. In addition, soluble ICs prepared *in vitro* had more potent inhibitory activity than IVIG in a mouse ITP model [9]. Furthermore, an Fc trimer with an artificially designed structure was shown to have enhanced potency over IVIG [10].

Although IVIG is a well-established therapeutic option for the treatment of various autoimmune and inflammatory diseases, the standard dose prescribed for autoimmune diseases, including ITP, is 2 g/kg, which is markedly high. To minimize the risk for thromboembolic events, slow IVIG administration has been recommended, as follows: 2 g/kg over 5 days, i.e. 0.4 g/kg/day in no less than 8 h each day [11]. The long time required to administer IVIG is burdensome to the medical staff and patients. Moreover, the use of IVIG has continued to increase in recent years, creating a gap between the demand and supply of IVIG [12]. Thus, there is a high unmet need for developing substitution therapies for IVIG with non-inferior efficacy at a reduced dose. Recombinant Fc multimers such as CSL730, a recombinant Fc trimer, and PF-06755347, a recombinant Fc multimer, have demonstrated high potency in preclinical studies and are under phase I development [13].

One of the major challenges in evaluating therapeutic candidates with a FcγR blockade mechanism is the development of animal models that can predict drug efficacy in humans. Although mouse ITP models have been used to investigate the causes of human ITP and mechanisms of action of IVIG, there are critical differences in the FcγRs of mice and humans [14, 15]. Previously reported mouse ITP models are induced by anti-platelet antibodies of either mouse IgG2a/IgG2b or IgG1 isotype, resulting in murine FcγRIV or FcγRIII dependent pathogenesis [14]. While murine FcγRIV is reported to be the ortholog of human CD16a, there are no reports of human ortholog of murine FcγRIII. Furthermore, the ortholog of human CD32a has not been identified in mice [14]. In humans, not only CD16a but also CD32a have been suggested to play important roles in ITP pathogenesis [3, 4]. Therefore, it is difficult to extrapolate the efficacy of new drug candidates in humans directly from the results obtained in mouse ITP models.

We previously found that the anti-haptoglobin (Hp) antibodies in IVIG generated ICs when mixed with serum and hypothesized that these antibodies were one of the effective components of IVIG. To replace them with monoclonal antibodies, we generated different types of anti-Hp monoclonal antibodies (unpublished data). The human Hp is a serum glycoprotein in which two or more α–β subunits are bound to each other by forming a disulfide bond between the α chains [16]. Since α–β subunits form multimers, we expected that anti-Hp monoclonal antibodies have the ability to form complexes with human Hp, which contains multiple IgG molecules. In this study, we characterized two anti-Hp monoclonal antibody clones, both of which formed an IC with Hp and bound more strongly to CD16a and CD32a compared to IgG monomers. We also generated defucosylated and Fc amino acid-modified anti-Hp antibodies for increased FcγR binding and evaluated their efficacy using an antibody-dependent cellular cytotoxicity (ADCC) inhibition assay.

In addition, we aimed to establish a novel cynomolgus monkey ITP model using the anti-platelet monoclonal antibody clone 26.4. Clone 26.4 has been identified as the maternal antibody against glycoprotein IIIa (GPIIIa) in fetal and neonatal alloimmune thrombocytopenia [17]. It is also reported that the most common subclass of anti-platelet antibodies in chronic ITP is IgG1 [18]. We expected thrombocytopenia would be induced by 26.4 IgG1 in cynomolgus monkeys.

To confirm the efficacy of the Fc-modified anti-Hp antibody, we administered the antibodies and analyzed the platelet count. Results showed that the Fc-modified anti-Hp antibody had exerted non-inferior efficacy compared to IVIG at a considerably lower dose. We also monitored the receptor occupancy (RO) of CD16a on natural killer (NK) cells in the peripheral blood to evaluate the pharmacodynamics of the anti-Hp monoclonal antibody and IVIG.

## Materials and methods

### Preparation of recombinant antibodies

The complementary DNA (cDNA) coding variable regions of both heavy and light chains of anti-Hp antibodies (unpublished data) were inserted into the human IgG1 expression vector. The constructs were then transfected into Chinese hamster ovary (CHO) cells and purified from supernatants with Protein A-conjugated Sepharose columns (ProteNova). The recombinant antihuman GPIIIa (CD61) antibody (clone 26.4) [17] was also prepared as described above. In order to prepare defucosylated anti-Hp antibodies, the recombinant vector was introduced into fucosyltransferase 8-knockout CHO cells [19]. Furthermore, we generated two types of defucosylated Fc amino acid-modified anti-Hp antibodies, D16 (defucosylated; CD16a high-affinity amino acid mutations, S239D and I332E) and D1632 (defucosylated; CD16a/CD32a high-affinity amino acid mutations, G236A, S239D, and I332E) [20]. The two types of synthetic DNA of the gene sequence of the Fc domain of human IgG1 in which amino acids were modified were inserted in the recombinant vector. Fc-modified negative control antibodies (anti-dinitrophenyl [DNP] antibodies) were prepared based on a previously published method [21].

### CD16a binding assay by enzyme-linked immunosorbent assay (ELISA)

Anti-tetra-His antibodies (QIAGEN) dissolved in phosphate-buffered saline (PBS) were adsorbed onto a 96-well plate (Thermo Fisher Scientific). After blocking with 1% bovine serum albumin (BSA)–PBS, the soluble His-tagged CD16a were incubated in the plates at room temperature for 2 h. After washing with PBS containing 0.1% Tween 20 (wash buffer), a diluted mixed solution of the anti-Hp antibodies and the human Hp (Japan Blood Products Organization) in 1% BSA–PBS was incubated at room temperature for 2 h. The mixed solution was prepared by mixing the components in 1% BSA–PBS at a molar ratio of 1:2 and incubating for 1 h at room temperature. After washing with the wash buffer, bound antibodies were detected using goat antihuman IgG (H&L)-horseradish peroxidase (American Qualex) with ABTS. The reaction was stopped with 1% sodium dodecyl sulfate, and the absorbance at a sample wavelength of 415 nm and a reference wavelength of 490 nm was measured. IVIG (Venoglobulin IH 5% or Polyglobin-N 10%) was purchased from Japan Blood Products Organization. The soluble CD16a (158V allotype) with His-tag was prepared as described previously [22]. The effective concentration (EC_50_) value was calculated by measuring the IgG concentration that produced 50% of the maximum absorbance. These assays were performed in duplicate and were repeated once.

### Evaluation of inhibitory activity against the ADCC reaction using peripheral blood from healthy human donors

Defucosylated anti-CD20 antibody rituximab was prepared based on a previously described method [23]. The inhibitory activity of anti-Hp antibodies against the ADCC reaction was investigated using peripheral blood from healthy human donors as described previously [23]. First, the anti-Hp antibody, the anti-DNP antibody, the anti-human CD16 antibody (clone 3G8, Biolegend), and IVIG in RPMI 1640 were added to peripheral blood and incubated for 30 min at 37°C (5% carbon dioxide [CO_2_]) in a 48-well plate (Greiner Bio-One GmbH). Next, defucosylated rituximab was added. In non-treated wells, RPMI 1640 was added instead of the defucosylated rituximab. In no inhibition wells, RPMI 1640 was added instead of test samples. The 48-well plate was cultured for 20 h at 37°C (5% CO_2_), and the number of B cells was counted as follows: CountBright Absolute Counting Beads (Molecular Probes) were added, followed by hemolysis using red blood cell lysis buffer (eBioscience) according to the manufacturer’s instructions. After centrifugation, the supernatant was discarded, and the cells obtained as a residue were transferred to a 96-well plate with a U bottom (Falcon) and suspended in 1% BSA-PBS containing 0.05% sodium azide (NaN_3_) and 1 mmol/l ethylenediaminetetraacetic acid (EDTA). After treatment with the human FcR blocking reagent (Milteniy Biotec), cells were stained with allophycocyanin (APC) mouse anti-human CD19 (BD Biosciences) and phycoerythrin (PE) mouse anti-human CD2 (BD Biosciences). Dead cells were stained using the Live/Dead Fixable Violet Dead Cell Stain Kit (Molecular Probes). Data were acquired using BD Biosciences’ FACSCanto II and analyzed with the FlowJo™ (TreeStar Inc., Ashland, OR, USA) software. Live/Dead- CD19+ CD2- cells were defined as B cells. ADCC activity was evaluated by analyzing the number of B cells per 2,400 CountBright Absolute Counting Beads. All ADCC reactions were done in triplicate and were repeated twice.

### Cynomolgus monkey model of ITP

This experiment was conducted at Ina Research (Nagano, Japan). Non-treated male cynomolgus monkeys (age, 2 years; body weight, 2.05–2.72 kg) were included. Four monkeys were included in each group. HpmAb/D1632 at doses of 0.5, 2, 5, and 20 mg/kg was subcutaneously administered (day 0) (HpmAb1/D1632 group). IVIG at a dose of 1 g/kg was intravenously administered on two consecutive days (days 0 and 1) (IVIG group). The antibody storage buffer was subcutaneously administered to control animals (day 0) (Control group). After administration of IVIG or HpmAb/D1632, anti-platelet antibody at a dose of 0.15 mg/kg was intravenously administered on day 1 to induce thrombocytopenia. Blood samples were collected from the femoral vein, and the number of platelets was counted using the Sysmex XN-200 Hematology System (Sysmex, Hyogo, Japan) before (on days 0 and 1) and after (on days 2, 3, 5, and 8) thrombocytopenia induction. The platelet count before administration of IVIG or HpmAb/D1632 on day 0 was defined as the baseline platelet count. The rate of residual platelets was calculated in comparison to that of the baseline (day 0) using the following equations:


$$\begin{array}{ll} The\;rate\;of\;residual\;platelets= & \;[the\;platelet\;count\;\left(at\;each\;time\;point\right)\\ & /the\;platelet\;count\;\left(baseline\right)]\times 100 \end{array}$$

$$\begin{array}{l} The\;rates\;of\;residual\;platelets\;for\;each\;group\;\left(n=\;4\;per\;group\right)\\ \;are\;expressed\;as\;themean\pm standard\;error\;\left(SE\right) \end{array}$$

All experiments were performed in accordance with the Standards for Proper Conduct of Animal Experiments at Kyowa Kirin Co., Ltd., under the approval of the company’s Institutional Animal Care and Use Committee or the Regulations for Animal Experiments. Kyowa Kirin Co., Ltd., is fully accredited by the Association for Assessment and Accreditation of Laboratory Animal Care International.

### Monitoring CD16a occupancy on NK cells

The expression level of occupied and unoccupied CD16a on NK cells was evaluated as follows: Blood samples for this assay were collected on days 0, 1, 2, 3, 5, and 8. PE anti-human CD45 (BD Bioscience), PE-Cy7 anti-CD159a (Beckman Coulter), FITC anti-human CD14 (BD Bioscience), APC anti-human CD16 (clone 3G8; BD Bioscience), and Alexa Fluor 647 anti-human CD16 (clone DJ130c; AbD Serotec) were used. Both occupied and unoccupied CD16a were detected using clone DJ130c, and unoccupied FcγRIII was detected using clone 3G8, which competes with IgG [24].

FACS Lysing solution (BD Bioscience) was added to the blood samples and the samples were incubated for 15 min to prevent internalization of detection antibodies. PBS supplemented with 2% fetal bovine serum (FBS), 0.05% NaN_3_, and 1mM EDTA (FCM buffer) was added, and cells were pelleted by centrifugation. The cell pellet was suspended and incubated with appropriate antibodies or isotype controls for 30 min in U-bottomed plates. Data were acquired as described above. The expression level of occupied CD16a on NK cells (CD45+CD14−CD159a+; referred to as RO) and unoccupied CD16a on NK cells (referred to as free ratio) at each time point was calculated in comparison to that of the baseline (day 0) using the following equations:


$$\begin{array}{ll} \%\;Free\;ratio\;= & \;[\left(geo\;MFI\;of\;3G8-geo\;MFI\;of\;isotype\right)\;\\ & \left(at\;each\;time\;point\right)/(geo\;MFI\;of\;3G8\\ & -geo\;MFI\;of\;isotype)\;\left(baseline\right)]\times 100 \end{array}$$

$$\begin{array}{l} \%\;RO\;=\;\left[1-RO\;\left(at\;each\;time\;point\right)/(RO\;\left(baseline\right)\right]\times 100 \end{array}$$

$$\begin{array}{ll} & \%\;RO\;\left(at\;each\;time\;point\right)\;=[\left(geo\;MFI\;of\;3G8-geo\;MFI\;of\;isotype\right)\\ & \;\left(at\;each\;time\;point\right)/(geo\;MFI\;of\;DI130c\\ & -geo\;MFI\;of\;isotype)\;\left(at\;each\;time\;point\right)\}\times 100 \end{array}$$

$$\begin{array}{ll} & \%\;RO\;\left(baseline\right)\;=[\left(geo\;MFI\;of\;3G8-geo\;MFI\;of\;isotype\right)\\ & \;\left(baseline\right)/\left(geo\;MFI\;of\;DI130c-geo\;MFI\;of\;isotype\right)\;\\ & \left(baseline\right)\}\times 100 \end{array}$$

Geo MFI refers to the geometric median fluorescence intensity.

### Statistical analysis

Statistical analyses were performed using the statistical analysis software (SAS; SAS Institute, Cary, NC, USA). Significant differences between the experimental groups were determined by one-way analysis of variance, followed by the Tukey–Kramer multiple comparison test. A *P*-value of < 0.05 or < 0.01 was predetermined as the criterion for statistical significance.

## Results

### Binding activity of anti-Hp antibodies to CD16a and CD32a was improved by IC formation

**Figure 1:**
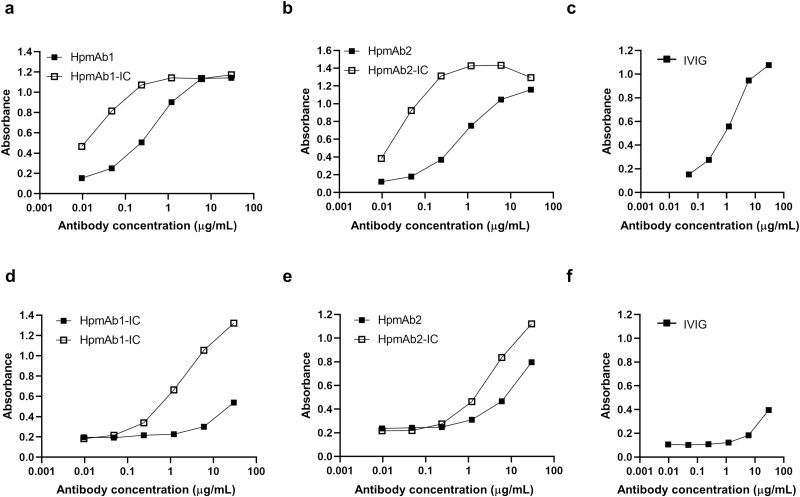
Binding activity to CD16a and CD32a (ELISA). (**a**) and (**b**) show the binding activity of two anti-Hp antibody clones to CD16a in the absence or presence of human Hp. (**c**) shows the binding activity of IVIG to CD16a. (**d**) and (**e**) show the binding activity of the two monoclonal antibodies to CD32a. (**f**) shows the binding activity of IVIG to CD32a. The horizontal axis represents antibody concentration (μg/ml), and the vertical axis represents absorbance (415–490 nm). ■ indicates the results of the anti-Hp antibody alone, and □ indicates the results after forming an IC. ELISA was performed in duplicate. The symbols indicate the mean. ELISA = enzyme-linked immunosorbent assay; Hp = haptoglobin; IC = immune complex; and IVIG = intravenous immunoglobulin.

Two clones, HpmAb1 and HpmAb2, were selected, and recombinant antibodies were prepared as human IgG1 and kappa LC (HpmAb1/wild-type (WT) and HpmAb2/WT). Fig. 1a and b shows the binding activity of HpmAb1 and HpmAb2 to CD16a, respectively, in the presence or absence of Hp. In the absence of Hp, the EC_50_ values for HpmAb1 and HpmAb2 were 0.405 and 0.763 μg/ml, respectively, which were comparable to the EC_50_ value of 1.06 μg/ml for IVIG (Fig. 1c). Furthermore, the EC_50_ values for HpmAb1 and HpmAb2 in the presence of Hp were 0.0540 and 0.0490 μg/ml, respectively, approximately 10-fold lower than when Hp was absent (Fig. 1a and b). These results suggest that when antibodies are mixed with human Hp, which consists of at least two subunits, IC formation is induced, and hence, the binding activity against CD16a is improved by the avidity effect. We found that the binding activity of these two clones to CD32a was also improved by IC formation (Fig. 1d and e).

### Fc-modified anti-Hp antibody exhibited high inhibitory activity against the ADCC reaction by forming ICs in the blood

**Figure 2:**
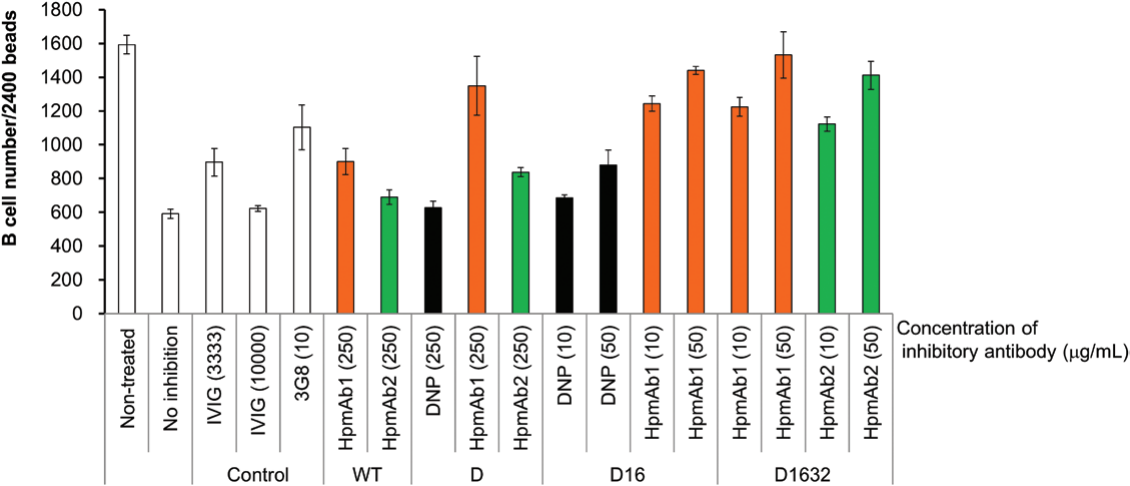
Inhibitory activity of Fc-modified anti-Hp antibodies against the ADCC reaction using peripheral blood from healthy human donors. The inhibitory activity of various modified versions of antibodies, HpmAb1, HpmAb2, and anti-DNP antibodies, against the ADCC reaction is shown in comparison with IVIG. The test samples and defucosylated rituximab were added to normal donor peripheral blood and these were incubated (*n* = 3). The vertical axis shows the number of B cells per 2400 CountBright Absolute Counting Beads. Data are from triplicates mean ± SD. 3G8 = antihuman CD16 antibody; ADCC = antibody-dependent cellular cytotoxicity; D = defucosylated; D16 = defucosylated and CD16a high-affinity Fc amino acid mutation; D1632 = defucosylated and CD16a/CD32a high-affinity Fc amino acid mutation; DNP = dinitrophenylhydrazine; IVIG = intravenous immunoglobulin; and WT = wild type.

To validate the FcγR-blocking activity of anti-Hp antibodies, we evaluated the inhibitory activity of the antibodies against the ADCC reaction using whole blood. In addition, we prepared various versions of Fc-modified (D = defucosylated; D16 = defucosylated and CD16a high-affinity Fc amino acid mutation; D1632 = defucosylated and CD16a/CD32a high-affinity Fc amino acid mutation) anti-Hp antibodies, and the enhanced binding to CD16a and CD32a was confirmed by Biacore or ELISA assays (data not shown). Next, we evaluated whether the inhibitory activity was enhanced by increased FcγR binding (Fig. 2). B-cell numbers were restored by treatment with anti-CD16 antibody (3G8), suggesting that the inhibitory activity against CD16-dependent ADCC is detectable in this assay. Anti-Hp antibodies modified with either defucosylation alone (D) or with both defucosylation and CD16a high-affinity Fc amino acid mutation (D16) exhibited comparable or higher inhibitory activity than anti-DNP antibodies with the same Fc modification. Further, compared to IVIG at very high concentrations, anti-Hp antibody with any Fc exhibited comparable or higher ADCC inhibition at lower concentrations. These results suggest that anti-Hp antibodies could form ICs with Hp in human peripheral blood, which resulted in stronger CD16a blockade compared to that with anti-DNP antibodies or IVIG. Also, Fc-modified (D16 or D1632) anti-Hp antibodies, when compared to wild-type anti-Hp antibody, exhibited comparable or higher inhibitory activity at lower concentrations (Fig. 2). These results suggest that stronger CD16a binding by Fc modification leads to higher ADCC inhibitory activity.

### Thrombocytopenia was prevented by IVIG and Fc-modified anti-Hp antibody in the cynomolgus monkey model

**Figure 3:**
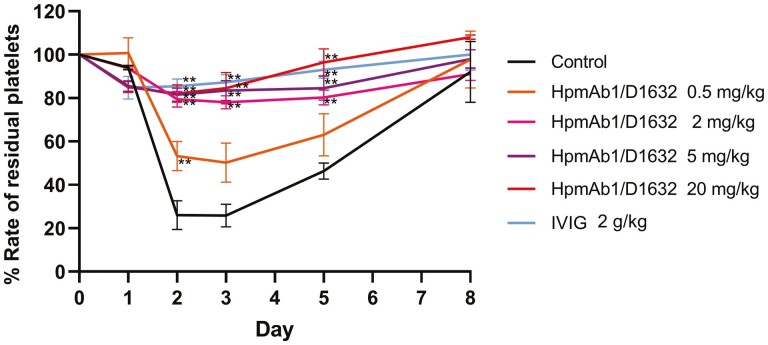
*In vivo* efficacy of HpmAb/D1632 in a monkey model of ITP. The rate of residual platelets as effect of IVIG and HpmAb1/D1632 on prevention of ITP induction is shown. HpmAb/D1632 at doses of 0.5–20 mg/kg was subcutaneously administered (day 0). IVIG at a dose of 1 g/kg was intravenously administered on two consecutive days (days 0 and 1). After administration of HpmAb/D1632 or IVIG, intravenous injection of the anti-platelet antibody was administered on day 1. The rates of the four monkeys in each group were expressed as the mean ± SE. Double asterisks indicate statistically significant differences in comparison with the control group (*P* < 0.01). D1632 = defucosylated and CD16a/CD32a high-affinity Fc amino acid mutation; ITP **=** immune thrombocytopenic purpura; IVIG = intravenous immunoglobulin; SE = standard error.

To evaluate the effect of Fc-modified anti-Hp antibody in an *in vivo* model, we established a monkey model of ITP. After anti-platelet antibody administration on day 1, the rate of residual platelets in the control group decreased to 25.8% on day 3 (Fig. 3). The rate then gradually recovered and reached up to 92% on day 8. When prophylaxis treatment was provided with a high dose of IVIG, residual platelets were maintained at a high level during the test period. To administer 2 g/kg of IVIG, the dose used in the treatment of ITP, 1 g/kg of IVIG was administered on two consecutive days (days 0 and 1). On the other hand, almost complete prevention of thrombocytopenia was achieved by a single subcutaneous administration of HpmAb1/D1632 (day 0). A dose of 0.5 mg/kg partially inhibited thrombocytopenia (the rate of residual platelets on day 3 was 50.3%), and doses above 2 mg/kg inhibited thrombocytopenia to levels comparable to those in the IVIG group. It was demonstrated that HpmAb1/D1632 was 100-fold more potent than IVIG in this model.

### Expression level of unoccupied CD16a on NK cells was downregulated by IVIG treatment and Fc-modified anti-Hp antibodies

**Figure 4:**
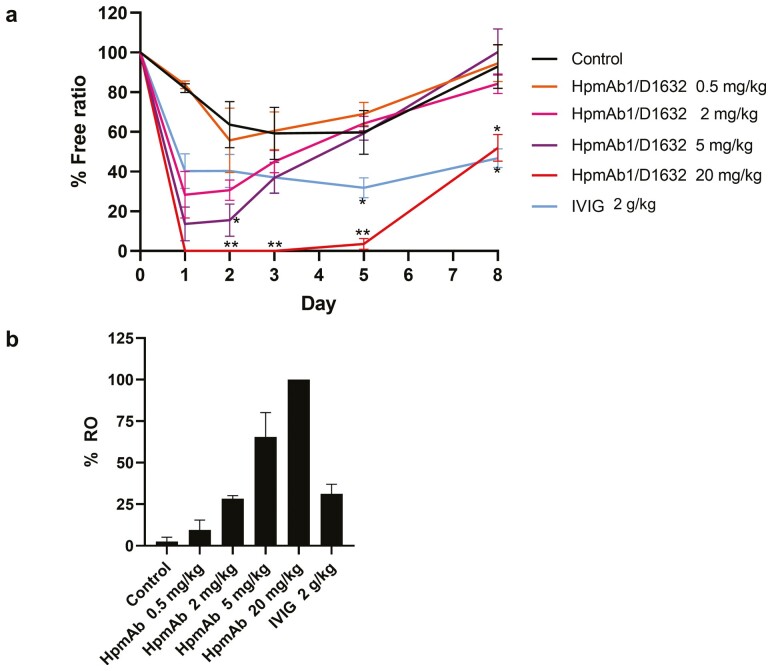
Effect of HpmAb/D1632 on CD16a occupancy on NK cells in a monkey model of ITP. Free ratio (**a**) and RO (**b**) as effects of IVIG and HpmAb1/D1632 on CD16a occupancy are shown. HpmAb/D1632 (0.5–20 mg/kg) was administered subcutaneously (day 0). IVIG (1 g/kg) was administered intravenously on two consecutive days (days 0 and 1). After administration of HpmAb/D1632 or IVIG, thrombocytopenia was induced by intravenous injection of the anti-platelet antibody on day 1. The rates of the four monkeys in each group were expressed as the mean ± SE. One asterisk and double asterisks indicate statistically significant differences in comparison with the control group (*P* < 0.05 and *P* < 0.01, respectively). D1632 = defucosylated and CD16a/CD32a high-affinity Fc amino acid mutation; ITP **=** immune thrombocytopenic purpura; IVIG = intravenous immunoglobulin; NK = natural killer; RO = receptor occupancy; SE = standard error.

To investigate whether the mechanism of action of IVIG and anti-Hp monoclonal antibodies is FcγR dependent, we evaluated FcγR occupancy using peripheral blood. No suitable tool antibodies for detecting unoccupied CD32a have been reported; on the other hand, anti-human CD16 antibody (clone 3G8) has been reported to compete with the Fc domain of IgG, thus enabling detection of unoccupied CD16 only [24]. Therefore, we established the method to monitor the expression level of unoccupied CD16a on NK cells (free ratio) and that of occupied CD16a (RO) on NK cells by staining with two CD16 antibodies, Fc-competing 3G8 and non-competing DJ130c. Fig. 4a shows the result of the free ratio. In the control group, the free ratio partially decreased to 63.6% by day 2 and remained at around 60% until day 5; it recovered to 92.9% on day 8. These results suggest that the free ratio may slightly decrease by injecting antiplatelet antibodies. The free ratio in the IVIG group decreased to 40.3% on day 1, and it was maintained at a similar level during the test period. In the HpmAb1/D1632 group, a dose-dependent decrease in the free ratio was observed. At doses of 2 and 5 mg/kg, the free ratio decreased to a level lower than that in the IVIG group on day 1 but started to recover on day 3 and reached the same level as that in the control group by day 5. At a dose of 20 mg/kg, the free ratio was 0% in all individuals on day 1; the level remained very low until day 5 and began to recover on day 8, but it was comparable to the day 8 level in the IVIG group. The CD16a RO on NK cells was also evaluated, and a dose-dependent increase was observed in the HpmAb1/D1632 group, reaching 100% RO at 20 mg/kg, the highest dose (Fig. 4b). IVIG at 2 g/kg showed RO levels comparable to those of HpmAb1/D1632 at 2 mg/kg. Although the free ratio of HpmAb1/D1632 at a dose of 0.5 mg/kg was not decreased compared to that in the control group, a slight increase in RO was detected.

## Discussion

FcγRs have been elucidated to have a key role in the pathogenesis of several important autoimmune diseases. Therefore, many therapeutic agents targeting FcγR have been developed [25]. For example, GMA161, an anti-CD16 antibody, was shown to improve platelet counts in patients who did not respond to standard therapy (two out of four patients with ITP had improved platelet counts) [5]. ICs prepared by mixing mouse anti-human IgG antibodies and human Fc fragments have also shown more potent inhibitory activity than IVIG in a murine FcγRIII-dependent ITP model [9]. Two products of artificially designed Fc multimers that are under development in phase I have been reported to inhibit thrombocytopenia in two different murine ITP models (murine FcγRIII dependent and FcγRIV dependent) [10, 13]. In this study, we showed that simple monoclonal anti-Hp antibody exhibit very high FcγR-blocking ability by forming ICs with Hp in human blood. In terms of structural homogeneity and mass production, this novel monoclonal antibody is considered to be more beneficial than human Fc fragments cross-linked artificially.

IgG autoantibodies have been reported to induce immune responses against self-tissues through FcγRs, including CD16a and CD32a, on the surface of effector cells in some autoimmune diseases, such as ITP and systemic lupus erythematosus [3, 25, 26]. We hypothesized that a strong blockade of both receptors would be important for treating such autoimmune diseases. The results of *in vitro* experiments indicate that the binding of anti-Hp antibodies to CD16a and CD32a was enhanced by the addition of Hp, suggesting that the enhanced binding of anti-Hp antibodies to FcγR is due to the avidity effect of forming multivalent ICs containing more than one antibody molecule. Analysis of the molecular weight of ICs by size-exclusion chromatography–multi-angle light scattering (SEC–MALS) indicates that the ratio of antibody and Hp molecules of the main component of ICs was estimated to be 2:2 when either human Hp or monkey Hp was used (data not shown).

Furthermore, we found that Hp-anti-Hp antibody IC was formed when anti-Hp antibody was added to human blood *ex vivo*. Anti-Hp antibodies exhibited higher inhibitory activity than anti-DNP antibodies with the same Fc modification. Based on these results, the anti-Hp antibody might exhibit high inhibitory activity by forming IC with Hp in the blood. The inhibitory activity of anti-Hp antibody against ADCC was markedly enhanced when defucosylation and Fc amino acid mutations were introduced, suggesting that strong binding of IC to FcγR with its Fc domain competitively prevented the binding of rituximab to FcγR. Regarding the comparison between D16 and D1632, we evaluated the anti-Hp antibody modified with D1632 in the *in vivo* model since CD32a might also be involved in the disease and D1632 showed higher inhibitory activity against CD32a binding (Supplementary Fig. S1).

Results of the clinical trials of GMA161 have shown that adverse events such as fever-chill-vomiting reactions might be caused by cross-linking CD16a [5]. Moreover, Fc pentamers activate the FcγRs downstream signaling, while Fc trimers do not enhance either the downstream signaling or cytokine release [10]. To evaluate the safety risk of anti-Hp antibody ICs generated *in vivo*, we performed an *ex vivo* cytokine release assay and confirmed the lower immunostimulatory activity of the IC formed with HpmAb1 than that of HpmAb2 in healthy donor blood (Supplementary Fig. S2). In addition, cytokine release of HpmAb2/WT was not enhanced by Fc modifications (D and D16), suggesting that the immunostimulatory activity of HpmAb1/D1632 is not expected to exceed that of HpmAb1/WT. Indeed, HpmAb1/D1632 induced only low levels of cytokine production, and no adverse events, including neutropenia and symptoms related to cytokine release syndrome, were observed after administration of 20 mg/kg or 100 mg/kg of HpmAb1/D1632 to cynomolgus monkey in safety studies (data not shown). Based on these results, the IC comprising Hp and HpmAb1was considered to have a structure suitable for blocking FcγR without eliciting any undesired responses. Because the number of antibody molecules in our IC was estimated to be two, these results are consistent with the finding that the Fc trimer does not activate FcγR [10].

The murine ITP models reported in studies of IVIG mechanisms are either FcγRIII mediated or FcγRIV mediated, depending on the Fc subclass of anti-platelet antibodies [13, 14]. However, either of the murine models are not suited for estimating the efficacy of our Fc-modified antibodies because the full efficacy of our antibodies is expected to be achieved by strong binding to human CD16a and CD32a, both by IC formation and Fc modification. As human or monkey effector cells have been utilized to accurately evaluate the effect of Fc modification [27], we established a cynomolgus monkey ITP model to predict the efficacy of our drug candidates. As per *in vivo* test, acute thrombocytopenia was induced in cynomolgus monkeys using an anti-platelet antibody cloned from human thrombocytopenia patients, and thrombocytopenia was prevented by IVIG administration. These results show the establishment of a primate (cynomolgus monkey) model of ITP was achieved for the first time in our study.

We evaluated the *in vivo* efficacy of HpmAb1/D1632 using the established monkey model. The results showed that HpmAb1/D1632 exhibited non-inferior efficacy compared to IVIG at considerably low doses. We evaluated FcγR occupancy at the same time to investigate the mechanism of action of IVIG and HpmAb1/D1632 in the cynomolgus monkey ITP model. Because it is difficult to evaluate FcγR occupancy on the surface of macrophages in the reticuloendothelial system where platelet phagocytosis occurs over time, we evaluated CD16a occupancy on NK cells in peripheral blood. We found that the free ratio on NK cells decreased and the RO on NK cells increased by IVIG and HpmAb1/D1632 treatment. As we observed a clear relationship between efficacy and occupancy, it was suggested that IVIG and HpmAb1/D1632 exerted efficacy by blockade of FcγRs on the surface of macrophages where platelet phagocytosis occurs. Our ADCC inhibition assay data also suggests that the high FcγR-blocking activity of HpmAb1/D1632 was a result of add-on effect of IC formation and Fc modification.

In conclusion, we generated Fc-modified anti-Hp monoclonal antibodies with high potency in FcγR-blocking activity. Furthermore, to the best of our knowledge, a monkey model of ITP was established for the first time in our study and using this model, we demonstrated that Fc-modified anti-Hp antibody has non-inferior efficacy compared to IVIG at considerably lower doses. In addition, blockade of FcγR was suggested to be one of the important mechanisms of IVIG and the anti-Hp antibody in the monkey model of ITP.

## Supplementary Material

uxac112_suppl_Supplementary_Figure_S1

uxac112_suppl_Supplementary_Figure_S2

uxac112_suppl_Supplementary_Material

## Data Availability

The datasets generated and/or analyzed during the current study are available from the corresponding author on reasonable request.

## Abbreviations

ADCC: antibody-dependent cellular cytotoxicity
APC: allophycocyanin
cDNA: complementary DNA
CHO: Chinese hamster ovary
CO_2_: carbon dioxide
DNP: dinitrophenylhydrazine
EC_50_: effective concentration
EDTA: ethylenediaminetetraacetic acid
ELISA: enzyme-linked immunosorbent assay
FBS: fetal bovine serum
Fc: fragment crystallizable
Geo MFI: geometric median fluorescence intensity
GPIIIa: glycoprotein IIIa
Hp: haptoglobin
IC: immune complex
IgG: immunoglobulin G
ITP: immune thrombocytopenic purpura
IVIG: intravenous immunoglobulin
NaN_3_: sodium azide
NK: natural killer
PBS: phosphate-buffered saline
PE: phycoerythrin
RO: receptor occupancy
SAS: statistical analysis software
SE: standard error
SEC-MALS: size-exclusion chromatography-multi-angle light scattering
