# Supplementary figures and images for: A novel monoclonal antibody with improved FcγR blocking ability demonstrated non-inferior efficacy compared to IVIG in cynomolgus monkey ITP model at considerably lower dose
Source: Clin Exp Immunol. 2022 Dec 8;211(1):23–30. doi: 10.1093/cei/uxac112 (PMC9993454; doi:10.1093/cei/uxac112)

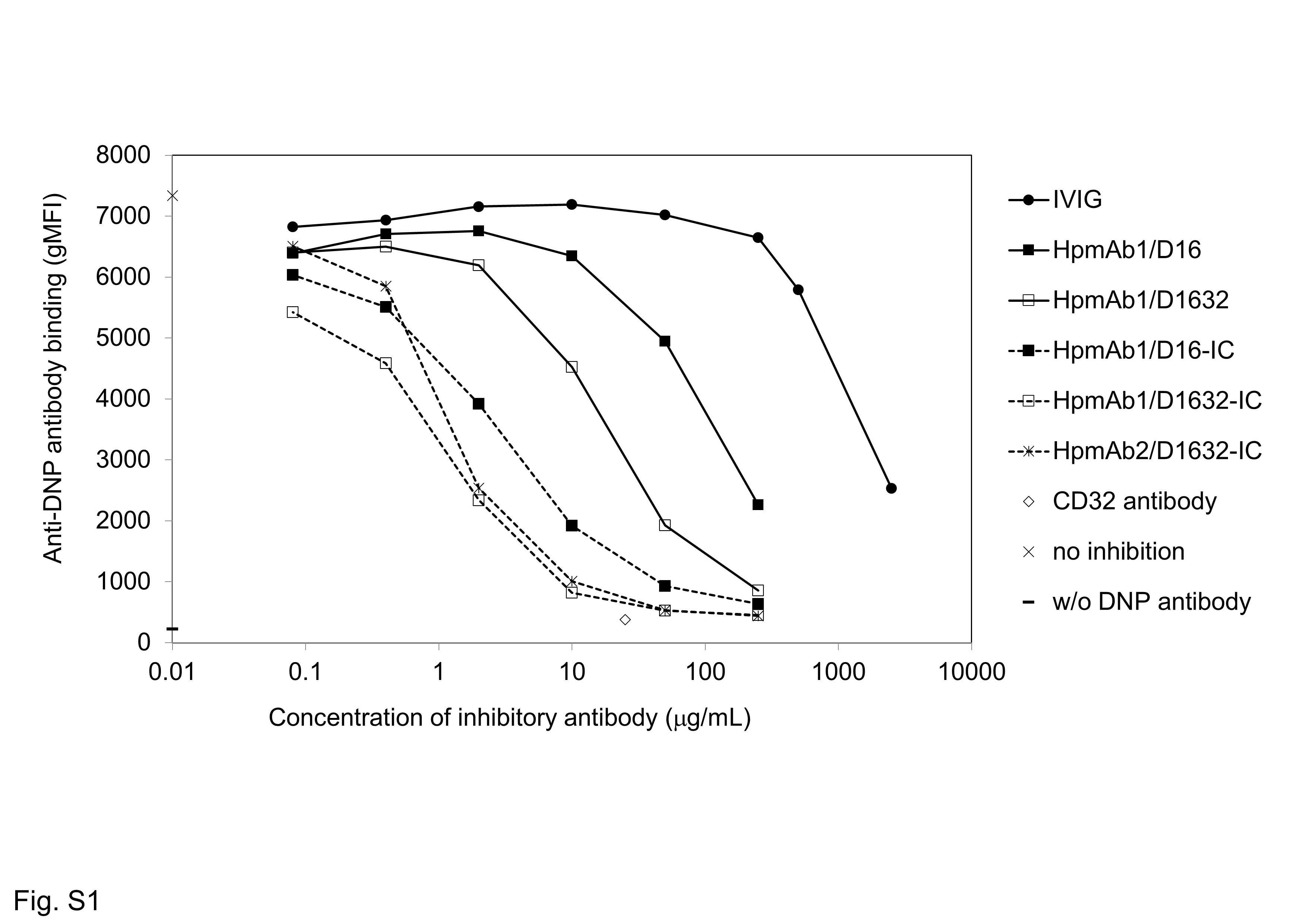

Supplement: uxac112_suppl_Supplementary_Figure_S1 [file uxac112_suppl_supplementary_figure_s1.jpeg]

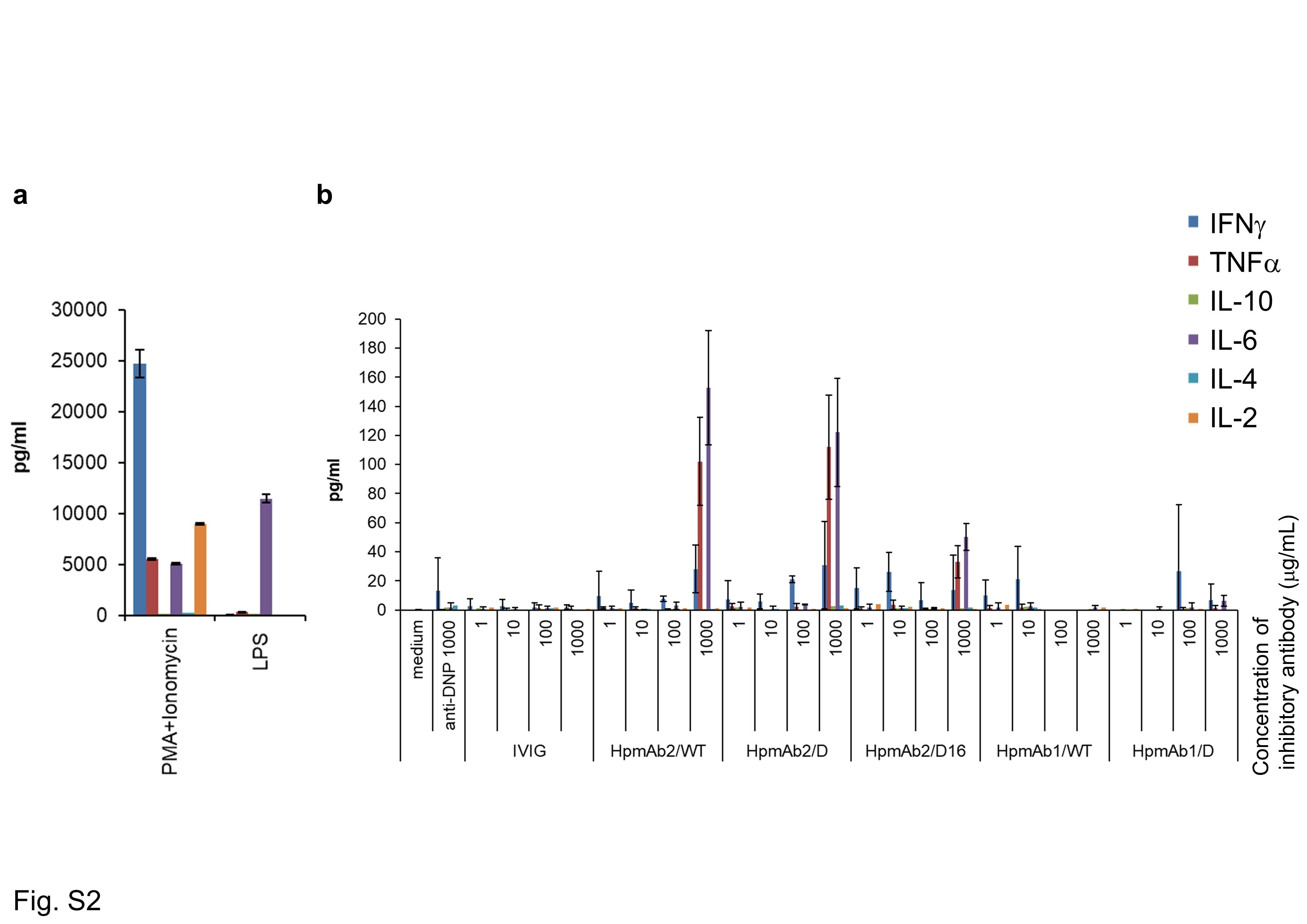

Supplement: uxac112_suppl_Supplementary_Figure_S2 [file uxac112_suppl_supplementary_figure_s2.jpeg]
