# Supplementary material for: A novel monoclonal antibody with improved FcγR blocking ability demonstrated non-inferior efficacy compared to IVIG in cynomolgus monkey ITP model at considerably lower dose
Source: Clin Exp Immunol. 2022 Dec 8;211(1):23–30. doi: 10.1093/cei/uxac112 (PMC9993454; doi:10.1093/cei/uxac112)
Supplement: uxac112_suppl_Supplementary_Material [file uxac112_suppl_supplementary_material.docx]

**Supplementary Supporting Information**

**Materials and methods**

**Evaluation of inhibitory activity against CD32a binding**

The inhibitory activity of Fc-modified anti-haptoglobin (Hp) antibodies against CD32a binding was investigated by using CD32a-expressing Chinese hamster ovary (CHO) cells. Fc-modified anti-Hp antibodies (in the absence or presence of human Hp) or intravenous immunoglobulin were evaluated as the test samples. The test samples and anti-dinitrophenylhydrazine (DNP) antibodies, preincubated with the DNP-BSA-Biotin conjugate, were added to CD32a-expressing cells suspended in FCM buffer and incubated in a 96-well plate. After washing with FCM buffer, the bound anti-DNP antibodies were detected by streptavidin, Alexa Fluor 647 conjugate (Molecular Probes). Data were acquired using BD Biosciences’ FACSCanto II and analyzed with the FlowJo™ (TreeStar Inc., Ashland, OR, USA) software. CHO cells expressing CD32a (GenBank accession code: AAH19931) were prepared internally. These assays were performed in two series and were repeated twice.

**Cytokine release assay using whole blood**

Immunostimulatory activity was assessed by measuring cytokine release after incubation with IVIG and anti- Hp antibodies in human peripheral blood. First, IVIG, or anti-Hp antibody diluted in RPMI 1640 were added to peripheral blood from healthy human donors and incubated for 24 hours at 37°C (5% carbon dioxide [CO_2_]) in a 96-well plate (Falcon). RPMI 1640 and anti-DNP antibody were used as negative controls. Phorbol 12-myristate-13-acetate (PMA)(50 ng/mL)(Sigma)/Ionomycin (1 μg/mL)(Sigma) and Lipopolysaccharide (LPS)(1 μg/mL)(Enzo Life Sciences) were used as positive controls. After incubation, the samples were centrifuged and supernatants were collected. Secreted cytokines in the samples were quantified using Cytometric Bead Array (CBA) Human Th1/Th2 Cytokine kit II (BD Bioscience) according to the manufacturer’s instructions. The cytokines measured were TNFα, IL-2, IL-4, IL-6, IL-10 and IFN-γ. Data were acquired using BD Biosciences’ FACS Canto II. The data were analysed with FCAP Array Software version 3 (Soft Flow, Inc., St. Louis Park, MN, USA). These assays were performed in triplicate and were evaluated using blood from four donors.
